# Supplementary material for: UV plasmonic properties of colloidal liquid-metal eutectic gallium-indium alloy nanoparticles
Source: Sci Rep. 2019 Mar 29;9:5345. doi: 10.1038/s41598-019-41789-8 (PMC6441023; doi:10.1038/s41598-019-41789-8)
Supplement: Supplementary file 1 — Supplementary file - text [file 41598_2019_41789_MOESM1_ESM.pdf]

Supplementary Information to:

UV plasmonic properties of colloidal liquid-metal eutectic gallium-indium alloy nanoparticles

By Philipp Reineck[a], Yiliang Lin[b], Brant C. Gibson[a], Michael D. Dickey[b], Andrew D. Greentree[a], and Ivan S. Maksymov[a, c]

[a] ARC Centre of Excellence for Nanoscale BioPhotonics, School of Science, RMIT University, Melbourne, VIC 3001, Australia

[b] Departments of Chemical and Biomolecular Engineering and Chemistry, North Carolina State University, Raleigh, NC 27695, USA

[c] Centre for Micro-Photonics, Swinburne University of Technology, Hawthorn, VIC 3122, Australia

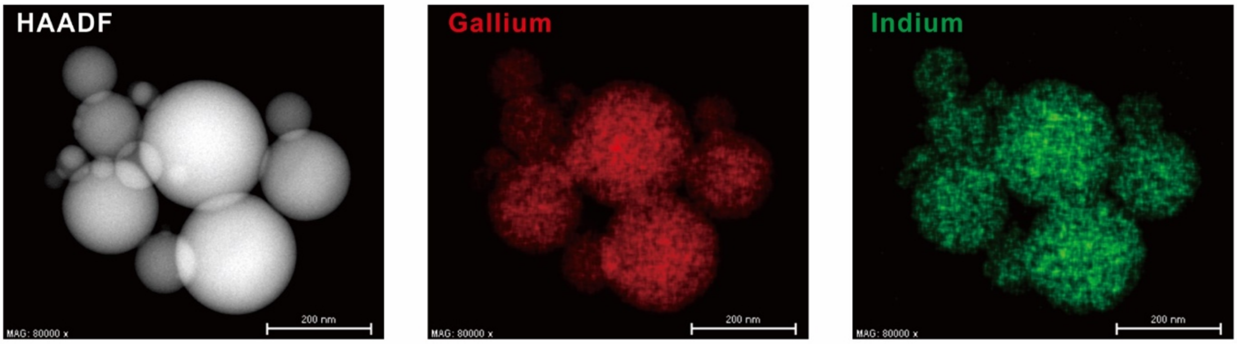

Figure S1. Energy dispersive X-ray spectroscopy (EDS) maps collected via high-angle annular dark-field scanning transmission electron microscopy (HAADF-STEM). The alloy used as a staring material is composed of 75 wt% gallium and 25 wt% indium and the eutectic composition of the synthesized nanoparticles is confirmed by the EDS maps above showing excellent colocalization of gallium and indium throughout all particles. For more details on the nanoparticle synthesis and characterization also see: Y. Lin, C. Cooper, M. Wang, J.J. Adams, J. Genzer, and M.D. Dickey. *Small* **11**, 6397 (2015).

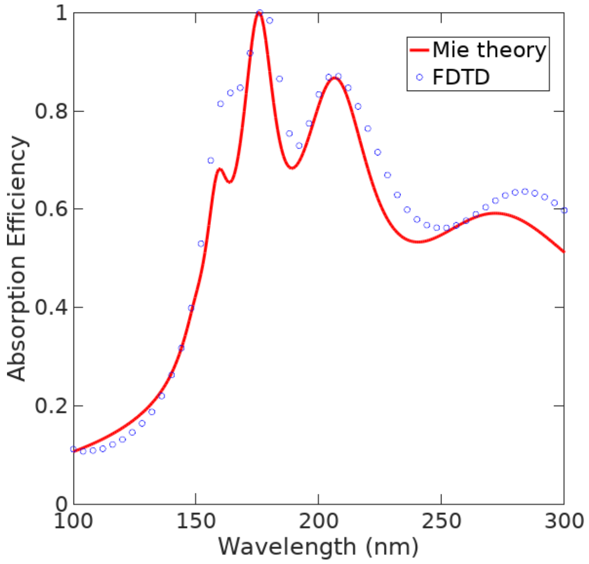

Figure S2. The optical absorption efficiency spectrum of the d = 100 nm single liquid-metal nanoparticle calculated using the exact Mie theory (solid line) and the finite-difference time-domain (FDTD) method (circles). Note that the gallium oxide layer is not taken into account. Both spectra are normalised to their peak value. In the FDTD method, a fine spatial resolution 0.5 nm was used and the simulation was run until a stringent convergence criterion was met.

FDTD Simulations

In our finite-difference time-domain (FDTD) simulations, we calculate the absorption spectrum of a spherical liquid-metal nanoparticle as $P_{abs} = -0.5\omega|\mathbf{E}|^2\mathrm{imag}(\varepsilon_{EGaIn})$, where ω is the angular frequency of the incident light, ε_{EGaIn} is the dielectric constant of the liquid-metal, and **E** is the electric field vector. An inherent part of the FDTD method is that each electric field component (E_x, E_y, E_z) is calculated at a different location within the finite difference mesh. Therefore, calculations of $|\mathbf{E}|^2$ are not trivial because E_x, E_y, E_z are not known at the same spatial location. Therefore, we interpolate all field components back to a common set of points (the origin of the finite difference mesh in our case). Despite this improvement and a high (0.5 nm) spatial resolution of the finite difference mesh, the FDTD method fails to reproduce all features seen in the spectrum produced by the exact Mie theory (Figure S2). This is well-known outcome because the plasmonic field is focused in a thin region inside the metal nanoparticle, thereby dramatically increasing the computational cost of FDTD simulations with high, Mie-theory-like accuracy. For more details on the FDTD also see: Taflove, A. & Hagness, S. C. Computational Electrodynamics: The Finite-Difference Time-Domain Method, 3rd ed. (Artech House, 2005).
